# The Evolution of Organosilicon Precursors for Low-k Interlayer Dielectric Fabrication Driven by Integration Challenges

**DOI:** 10.3390/ma14174827

**Published:** 2021-08-25

**Authors:** Nianmin Hong, Yinong Zhang, Quan Sun, Wenjie Fan, Menglu Li, Meng Xie, Wenxin Fu

**Affiliations:** 1 Key Laboratory of Science and Technology on High-Tech Polymer Materials, Institute of Chemistry, Chinese Academy of Sciences, Beijing 100190, China; nianminhong@iccas.ac.cn (N.H.); Yinongzhang@iccas.ac.cn (Y.Z.); iccassq@iccas.ac.cn (Q.S.); fanwenjie@iccas.ac.cn (W.F.); mengluli@iccas.ac.cn (M.L.); xiemeng20@mails.ucas.ac.cn (M.X.); 2 School of Chemistry and Chemical Engineering, University of Chinese Academy of Sciences, Beijing 100049, China

**Keywords:** low dielectric constant, dielectric interlayer, organosilicon, thin film, IC circuit, porous material

## Abstract

Since the application of silicon materials in electronic devices in the 1950s, microprocessors are continuously getting smaller, faster, smarter, and larger in data storage capacity. One important factor that makes progress possible is decreasing the dielectric constant of the insulating layer within the integrated circuit (IC). Nevertheless, the evolution of interlayer dielectrics (ILDs) is not driven by a single factor. At first, the objective was to reduce the dielectric constant (k). Reduction of the dielectric constant of a material can be accomplished by selecting chemical bonds with low polarizability and introducing porosity. Moving from silicon dioxide, silsesquioxane-based materials, and silica-based materials to porous silica materials, the industry has been able to reduce the ILDs’ dielectric constant from 4.5 to as low as 1.5. However, porous ILDs are mechanically weak, thermally unstable, and poorly compatible with other materials, which gives them the tendency to absorb chemicals, moisture, etc. All these features create many challenges for the integration of IC during the dual-damascene process, with plasma-induced damage (PID) being the most devastating one. Since the discovery of porous materials, the industry has shifted its focus from decreasing ILDs’ dielectric constant to overcoming these integration challenges. More supplementary precursors (such as Si–C–Si structured compounds), deposition processes (such as NH_3_ plasma treatment), and post porosity plasma protection treatment (P4) were invented to solve integration-related challenges. Herein, we present the evolution of interlayer dielectric materials driven by the following three aspects, classification of dielectric materials, deposition methods, and key issues encountered and solved during the integration phase. We aim to provide a brief overview of the development of low-k dielectric materials over the past few decades.

## 1. Introduction

Gordon Moore predicted, in the 1960s, that the number of transistors in a dense IC microprocessor within a modern electronic device would double every 18–24 months. This has been widely recognized as ‘Moore’s Law’ and, indeed, has held true for the past few decades [1]. Initially, this was done by shrinking the sizes of silicon chips to increase the number of transistors per wafer. However, starting from the early 2000s, this progression was limited by the fundamental properties of the materials used in fabricating these chips. Hence, the requirements for these materials are becoming increasingly delicate and finicky. Typically, modern IC chips consist of three main parts, semiconducting materials, including silicon, doped silicon, and other compounds that have similar properties (such as germanium); conductors, which are metals with high conductivity such as copper and aluminum; and insulators, which are placed between semiconductors and conductors as interlayer/barrier dielectrics to separate and protect the active area [2]. With the continuous scaling down of chip size dimensions and the technology node of ICs, the resistance-capacitance (RC) delay increases, leading to enhanced signal propagation delays, line-to-line crosstalk noise interference, and power dissipation to the invalidation of electronic devices. Therefore, the interconnecting insulator, usually called a low dielectric constant (low-k) interlayer, is becoming more and more important in order to reduce the RC delay, especially with the high frequency and high-speed signal transmission under the new circumstance of fifth-generation mobile communications technology (5G technology). Traditionally, silicon dioxide (SiO_2_) is the material of choice for this duty. However, the dielectric constant (k) of SiO_2_ is relatively high (k = 3.9–4.2), not meeting the demand of dielectric packaging materials for ultra-large-scale integrated circuits (ULSI) and 5G technology. Therefore, it is of great challenge to replace SiO_2_ with other promising candidates, e.g., organosilicons and porous organosilicons, to afford a lower dielectric constant layer (k < 3.9). Furthermore, the chemical vapor deposition (CVD) method has been adopted as mainstream in the formation of a high-quality, high-performance, low-k dielectric interlayer. Hence, the incorporation of organosilicon precursors with low processing temperature deposition is becoming more favorable by the semiconductor industry.

This review focuses on the progress of organosilicon dielectric materials from the original SiO_2_ to porous SiCOH materials, as well as integration challenges that occurred along with the incorporation of novel low-k materials and processing methods to overcome these challenges. Novel porous low-k materials have poor compatibility, are highly hydrophilic, and have worse physical, chemical, and thermal stabilities compared to traditional SiO_2_. All these features provided challenges for traditional integration processes. Therefore, after the discovery of porous low-k organosilicon materials, the focus was shifted to re-engineering the integration process. From such a perspective, the evolution of dielectric materials is a continuous process of combining chemistry progression and engineering refinement.

## 2. General Classification of Organosilicon Dielectric Material

### 2.1. SiO_2_

**Figure 1:**
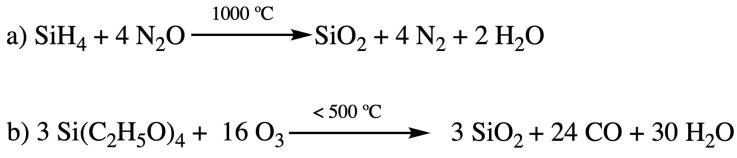
Deposition of SiO_2_ insulating layer with silane (**a**) and TEOS (**b**).

The SiO_2_ was initially deposited by a plasma-enhanced chemical vapor deposition (PECVD) technique with silane (SiH_4_) and N_2_O as precursors [2] (Figure 1a). The main disadvantage with this deposition method is the high fabrication temperature (usually up to 1000 °C) during integration. Such temperatures can easily disintegrate some of the previously deposited layers. Therefore, attempts were made to develop different chemistries and precursors to lower the temperature of the PECVD process [3]. Silicon alkoxide precursors soon had attracted people’s attention. The iconic ones include branched and cyclic silicon alkoxide such as tetraethylorthosilicate (TEOS) [4,5] and tetramethylcyclotetrasiloxane (TOMCATS^®^) [6]. They were used as alternative precursors for silane (SiH_4_) to lower the processing temperature (less than 500 °C) required for SiO_2_ deposition [2]. The reaction of silicon dioxide layer deposition with TEOS precursor is shown in Figure 1b. However, as the IC size shrunk to sub 180 nm dimension with the dielectric constant requirement of less than 3.0, silicon dioxide was destined to be replaced by other novel low-k materials, as shown in Table 1.

### 2.2. Silsesquioxane (SSQ) Based Materials

**Figure 2:**
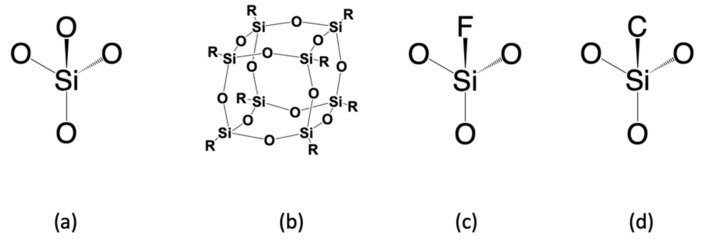
General classification of dielectric materials: (**a**) SiO_2_; (**b**) silsesquioxane (SSQ); (**c**) fluorinated silicon glass (FSG); (**d**) carbon-doped silica-based materials (SiCOH).

The semiconductor industry first attempted to replace SiO_2_ with SSQ materials. SSQ materials are members of organosilicon compounds with the chemical formula [RSiO_3/2_]_n_ (R = H, alkyl, aryl, or alkoxyl), usually adopting cage-like or ladder-like geometric structures. As a kind of typical SSQ material, polyoctahedral silsesquioxanes (POSS) have attracted lots of attention as preceramic polymer precursors to ceramic materials and nanocomposites, as demonstrated in Figure 2b. In general, alkyl and aryl groups exhibit lower dielectric constants and better mechanical properties than hydrogen atoms or alkoxyl. This is because their Si–C bonds, compared to Si–O and Si–H bonds, can create larger spatial volumes and lower polarizability. Such effects are demonstrated in Table 1, which indicates that methyl–Silsesquioxanes (MSSQs) generally have a lower dielectric constant (~2.7) than hydrogen–Silsesquioxane (~3.0). Manufacturing-wise, methyl–Silsesquioxanes have been examined to be suitable for spin-on technique fabrication [8,9,10]. Despite SSQ-based materials having attractive low dielectric constants (2.6–3.2), the integration issues (due to their low thermal stability and weak mechanical strength) have ruled them out from the semiconductor industry’s production line [11]. For example, hydrogen-terminated SSQ materials (HSSQ) are thermally unstable at 400 °C. Fully cured MSSQs show intrinsic dielectric constants between 2.7 and 2.9, thermal stability up to at least 500 °C, low moisture absorption, and attractive adhesion to metals. Unfortunately, the reduced network connectivity dramatically diminishes the mechanical properties compared to silicon dioxide. For example, Sang et al. reported that the hardness for a 100 nm thick MSSQ film only ranges from 0.29 to 0.54 GPa [12].

### 2.3. Silica-Based Materials

Silica-based materials have been successfully integrated into micro-transistors due to their outstanding chemical and thermal stabilities [11]. Such materials have the tetrahedral shape of silicon dioxide (Figure 2c,d). The density of different silica-based materials is usually between 2–3 g/cm^3^ [8]. Compared to silicon dioxide, such materials replace the high-polarity Si–O bonds with low-polarity Si–F bonds and Si–C bonds. The first generation of low-k materials applied in the semiconductor industry was fluorinated silicon glass (FSG), fabricated by chemical vapor deposition of TEOS, oxygen, and SiF_4_. The dielectric constant of FSG was significantly lower than SiO_2_ due to its less polarizable Si–F bonds. Furthermore, fluorine-containing organic groups such as CF_3_ are hydrophobic, which can protect silica from moisture contamination. This low-k material was used as a 0.18 μm technology node with the dielectric constants ranging from 3.5 to 3.8, depending on the molar ratio of the Si–F bond [13,14]. Generally, to fabricate FSG ILDs, a TEOS precursor would be used as the Si–O source, and a SiF_4_ precursor would be used as the Si–F source. Unfortunately, due to the presence of weak Si–F bonds, fluorine-doped silica materials do not possess sufficient thermal stability under high temperatures, not suitable for later integration steps. Moreover, neither silicon dioxide nor fluorinated silicon glass fit in the category of organosilicon materials. Their detailed electrical and mechanical properties have been discussed in a previous publication [8], which will not be included here.

The second generation of silica-based low-k materials is carbon-doped silica-based materials (SiCOH). As the structure in Figure 2d suggested, the SiCOH materials are produced by introducing lower polarity Si–C bonds into the tetrahedral silicon dioxide matrix. As shown in Table 1, these materials show excellent dielectric properties with k ranges from 2.7–3.3, depending on the concentration of carbon present in the deposited ILD. Moreover, the CH_3_ groups enlarge the inter-atomic distance of silica, which allows more free volume to further decrease the dielectric constant [11,15]. The existing SiCOH ILDs have been successfully integrated into 130 nm and 90 nm products [11].

**Figure 3:**
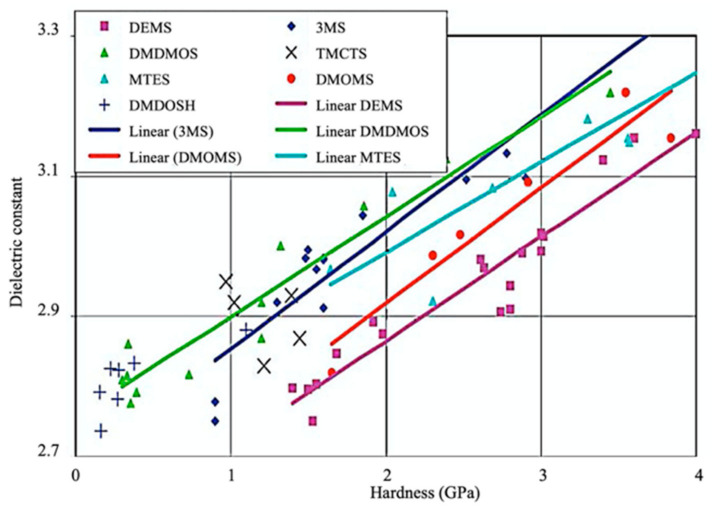
Dielectric constant vs. hardness for different precursors [2].

**Figure 4:**
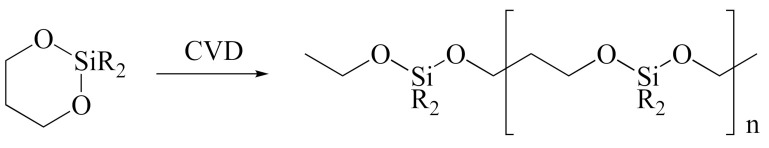
Ring-opening and expansion of DMDOSH.

The fabrication of silica-based ILDs requires a high concentration of Si–C bonds within their matrix. Therefore, precursors with one or more Si–C bonds were developed to meet such requirements. During the CVD step, these precursors are incorporated, with a certain ratio of oxidant precursors (such as O_2_, O_3_, N_2_O, or NO), under an inert gas atmosphere to create a layer of low-k thin film containing Si–C, Si–O, and Si–H bonds. It is also possible to combine alkyl siloxane precursors with oxidant precursors together and create a single precursor that has all the required components. One important factor that should be noted is that all of the atoms being introduced into the CVD chamber are important during the CVD process. Therefore, it is important to choose precursors with only Si, O, N, and H atoms. Here are some of the iconic examples of SiCOH precursors, diethoxymethylsilane (DEMS) [16,17], methyltriethoxysilane (MTES) [18], dimethoxymethylsilane (DMOMS), tetramethylcyclotetrasiloxane (TMCTS or TOMCATS^®^), octamethylcyclotetrasiloxane (OMCTS), dimethyldioxosilycyclohexane (DMDOSH), dimethyldimethoxysilane (DMDMOS) [19], and trimethylsilane (3MS) [20,21]. Table 2 shows molar ratios of Si–C, Si–O, and Si–H bonds with a silicon atom within each precursor molecule, respectively. The hardness and dielectric constant of the final ILDs corresponding to these precursors is shown in Figure 3 [2]. According to Figure 3, DEMS shows an excellent dielectric constant and hardness over other precursors. In 2003, Vincent et al. fabricated a dielectric layer with a DEMS precursor, demonstrating a Young’s modulus of 16.5 GPa and hardness of 2.8 GPa, while that based on 3MS and DMDMOS only showed a Young’s modulus of 8.76 GPa and hardness of 1.44 GPa, and Young’s modulus of 6.68 GPa and hardness of 1.2 GPa, respectively (Table 3) [22]. Besides DEMS, ring-structured DMDOSH also gained a lot of interest in mechanical strength enhancement. The ring-opening of such a structure may facilitate crosslinking during the CVD processes [22] (Figure 4). In theory, any volatile molecule (boiling point below about 250 °C) containing silicon and organic groups can be used as a SiCOH precursor. Vincent et al. systematically summarized some of the iconic organosilicon precursors in his patent [22]. For the semiconductor industry, finding a suitable alkyl siloxane precursor requires the ability for film formation to have good electrical and mechanical properties, as well as commercial availability or low cost to manufacture.

Though SiCOH has excellent mechanical stability for later integration steps, the dielectric constant of such materials is bottlenecked at 2.6 [2,11]. Achieving an even lower dielectric constant requires the introduction of pores into the dielectric matrix. The pores are filled with air, as shown in Table 1, since air has the lowest dielectric constant of 1 [2].

### 2.4. Porous Organo–Silicon Materials

The semiconductor industry constantly demands ultra-low-k materials (k < 2.6). Any further reduction of the k value would require introducing a pore structure into the dielectric material. Such pore structures can be obtained by either constitutive or subtractive methods [23,24,25]. Constitutive porosity, according to the International Union of Pure and Applied Chemistry (IUPAC) nomenclature, refers to pores created during deposition of an ILD without any post-fabrication treatment, and the pore structure depends on the original, as-deposited arrangement. Both SSQ and silica-based ILDs can have constitutive porosity. The pores are created mainly from the arrangement of atoms during the CVD step, which will be covered in later sections. Subtractive porosity involves the selective removal of part of the material from prefabricated films. In other words, subtractive porosity is created by incorporating an additional porogen precursor during the deposition process. The porogen precursors are hydrocarbons containing at least one alkene, alkyne, strained epoxy group, or carbonyl functional group. Porogen precursors will eventually be removed during the post-deposition-curing process [26,27]. The curing process can be done either thermally [28] or using an electron beam or ultraviolet radiation [29,30]. Some SiCOH precursors can even have built-in organic porogens. The reactive functional groups within precursors can readily generate radicals under plasma conditions, which may aid the incorporation of organic phases into the matrix. Nguyen et al. and Vincent et al. have systematically categorized the most common SiCOH precursors with embedded porogen organic groups in their patents [22,31]. Table 4 shows some of the most iconic SiCOH precursors with build-in organic porogens. The main difference between precursors shown in Table 2 and Table 4 is that these organosilicon precursors have one or more porogen functional groups.

The pore diameter plays a very important role in ILD manufacturing. When the pore diameter gets too big, other layers of films deposited during the dual-damascene process, such as metal barrier layer material, can easily penetrate the dielectric layer and ruin its dielectric properties. Moreover, large pores also cause low hardness and elastic modulus on ILDs, which would bring difficulties for later integration. When the pore diameter gets too small, the pores would not be effective enough for lowering the dielectric constant. According to the definition of the International Union of Pure and Applied Chemistry (IUPAC), pore sizes are classified as micro- (*ϕ* < 2.0 nm), meso- (*ϕ*~2–50 nm), and macro-pores (*ϕ* > 50 nm). Generally, SiOCH films have a constitutive porosity of about 5–15%, with a pore size of about 1 nm [32]. For dielectric applications in microelectronic devices, the ideal pores are uniformly around 1–4 nm in size. The pore size is highly dependent on the size of the porogen precursor molecule [33]. Typically, SiCOH precursors with built-in organic porogens can only deposit films with very small pores (~1 nm) and relatively low pore density (15%). Subtractive porosity can be as high as 90%, and pore sizes vary from two to tens of nanometers [15]. Furthermore, the pore size and density are more controllable during fabrication.

In general, the dielectric films with subtractive porosity have better mechanical and electrical properties than those with constitutive porosities. However, constitutive porosity can retain higher carbon content after deposition, which has more plasma damage resistance. Grill et al. reported a comparative experiment on mechanical and electrical properties of ILDs deposited with a mixture of alkyl siloxane (DEMS), porogen precursor bicycloheptadiene (BCHD), and porogen-embedded organosilicate precursors [27] As shown in Table 5, the V2 and V4 films contain trimethylsilylmethyl dimethoxymethylsilane (TDMMS) additive to relief plasma-induced damage (PID), which will be discussed in the 3rd part of this review. From the comparative data between V1.a and V3, the V1.a shows superior electrical and mechanical properties with higher breakdown voltage, hardness, and adhesion. However, the low carbon content of V1.a, along with higher pore density and larger pore size, increased its plasma-induced damage ratio.

The choice of porogen precursor is essential for producing porous ultralow-k (pULK) ILDs with desired mechanical and electrical properties. In theory, any hydrocarbon containing unstable functional groups with sufficient volatility to enable delivery as a gas to the PECVD reactor could be used as a porogen [29,34]. Two of the most common functional groups on porogen precursors are the vinyl group and the three-membered epoxy group. Favennec et al. conducted a comparative experiment between three porogens, hexene, hexadiene, and cyclohexene oxide [35,36], with decamethylcyclopentasiloxane as the SiCOH precursor. Table 6 shows the results of the matrix/porogen ratio and dielectric constant of the three deposited films before and after porogen removal [33]. From the results, a cured ILD deposited with cyclohexene oxide has the lowest dielectric constant (k = 2.2).

**Figure 5:**
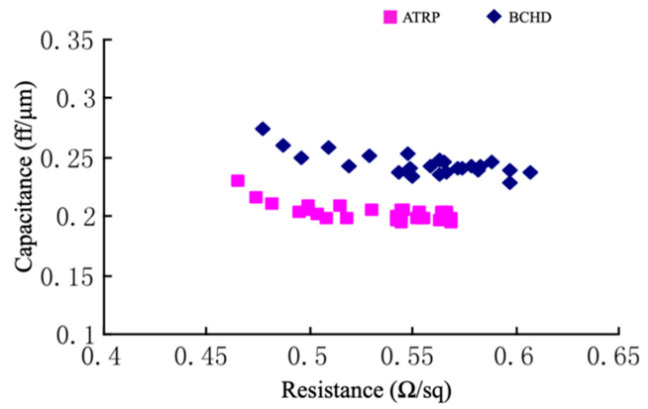
RC curve of ATRP and BCHD pULk films [25].

The industry has adopted α-terpinene (ATRP) [37] and bicycloheptadiene (BCHD) [38] as sacrificial porogens [27,29]. Both porogens have been successfully incorporated into a 28 nm technology node for pULK film manufacturing, with dielectric constants ranging from 2.4–2.6. Ming et al. reported a comparative study between porous thin films deposited with DEMS using ATRP and BCHD as porogens (Table 7). The data suggested that the hardness, Young’s modulus, and interfacial adhesion of the pULK film deposited with the ATRP porogen were 1.0 GPa, 7.1 GPa, and 5.9 J/m^2^, respectively, which were slightly higher than that deposited with BCHD (0.7 GPa, 5.1 GPa, and 5.4 J/m^2^). However, the pULK film fabricated with ATRP showed a slightly higher dielectric constant; the post-integration resistance-capacitance (RC) test results indicated that the k recovery effect of the ILD from the ATRP was better than that from the BCHD precursor. Figure 5 shows the RC curves of the post-integration pULK films from the ATRP and BCHD precursors, respectively. The pULK film from BCHD has a higher capacitance at the same resistance level compared to that from the ATRP precursor, indicating that the BCHD ILD was likely damaged by the plasma process during post-deposition integration [25].

**Figure 6:**
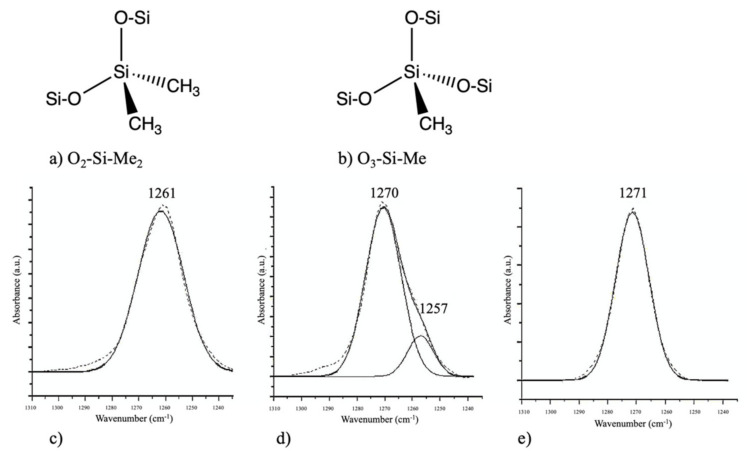
Top: schematic of two silicon matrixes, O_2_–Si–Me_2_ group (**a**) and O_3_–Si–Me group (**b**). Bottom: decomposition of Si–Me peak of FTIR spectra of DMCPS + porogen hybrid (**c**), DEMS + porogen hybrid (**d**), and DEMS + O_2_ + porogen hybrid (**e**) [39].

During the incorporation of SiCOH precursors with organic porogen precursors, it is important to consider if the removal of the porogen could cause any deformation of the ILDs. Favennec et al. studied the shrinkage of pULK SiCOH film after porogen removal. They chose decamethylcyclopentasiloxane (DMCPS) and DEMS as the SiCOH precursor and cyclohexene oxide as the organic porogen precursor. The DMCPS pULK film shrank by 33% after the thermal annealing (porogen removal) process, which led to a dielectric constant of 3.1. Such severe shrinkage resulted in no porosity on the final thin film. The DEMS pULK film, on the other hand, showed less than 20% shrinkage after annealing, which led to 13% porosity with a dielectric constant of 2.6. They further optimized the DEMS and cyclohexene oxide matrix by introducing an O_2_ precursor to increase the Si–O bond density within the matrix. As a result, the pULK film’s shrinkage decreased to less than 8%, and the porosity was increased to 25%, which further decreased its dielectric constant to 2.4. The exact reason for such results and the mechanism during fabrication remain unclear, but one possible explanation is the type of Si–O bonds within the pULK film matrix. As shown in Figure 6, O_2_–Si–Me_2_ (a) and O_3_–Si–Me (b) correspond to 1260 cm^−1^ and 1270 cm^−1^ in the FTIR spectrum, respectively. The FTIR peak position of Si–Me in DMCPS + cyclohexene oxide hybrid showed up at 1261 cm^−1^, suggesting that the Si–Me bonds are only in the O_2_–Si–Me_2_ configuration (Figure 6c). For the DEMS + cyclohexene oxide hybrid, the Si–Me signal can be split into two parts, a major contribution at 1270 cm^−1^ corresponding to O_3_–Si–Me and one minor at 1257 cm^−1^ corresponding to O_2_–Si–Me_2_ (Figure 6d). In the case of the DEMS + O_2_ + cyclohexene oxide hybrid, the Si–Me signal only appeared at 1271 cm^−1^, which corresponds to the O_3_–Si–Me configuration (Figure 6e). The additional Si–O bond within the O_3_–Si–Me tetrahedral shape might be a major factor for the film strength because it shares more similarities with the SiO_2_ crystalline structure. All the Si–O–Si structures within the network are crosslinked instead of linearly linked, which might, in turn, enhance the film’s strength and prevent it from collapsing after the annealing step [39]. 

Choosing the right ILD precursors can be very interesting because everything needs to come together in a harmonious way. During deposition, each atom and bond matters, and changing even the slightest factor could end up with drastically different results. The same principle may apply to deposition and integration as well.

## 3. Deposition Methods

**Figure 7:**
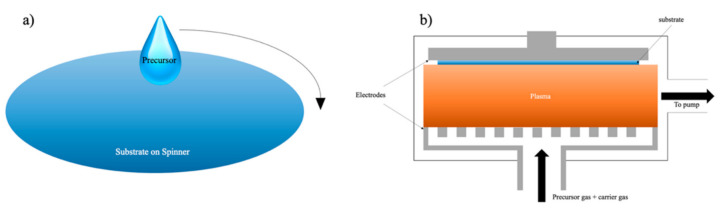
Schematic demonstration of spin-on deposition (**a**) and the PECVD method (**b**).

To understand why modifications would be needed on SiCOH precursors, we should first discuss the evolution of the insulating layer dielectric (ILD) deposition process and integration challenges over time. There are many ways to fabricate thin films, such as electroplating, dip coating, physical vapor deposition (PVD), atomic layer deposition (ALD), spin coating, and chemical vapor deposition (CVD). Electroplating is only suitable for depositing thin layers of noble metals such as copper, gold, and platinum. In the semiconductor industry, electroplating is frequently used for depositing copper wirings in the dual-damascene process, which will be covered in later sections. Dip coating works similar to spin-on coating except that the substrate will be completely submerged in the organosilicon solution, which brings difficulties for subsequent processes. The gentle conditions of physical vapor deposition do not change the chemical structure of the precursors, which can neither polymerize precursors nor turn them into a stable film. ALD is an ultra-thin film deposition technique that can uniformly deposit conformable films with a controlled thickness. However, the film growth rate of ALD is low because this method requires a longer period for chemical reactions of two or more precursors. Furthermore, it also has high material and energy waste during deposition. In IC manufacturing, two of the most common ways for fabricating low-k ILD are the sol-gel spin-on technique and CVD, which are usually enhanced by plasma [15]. Figure 7 shows the simplified demonstration of these two methods. 

### 3.1. Spin-On Deposition

**Figure 8:**
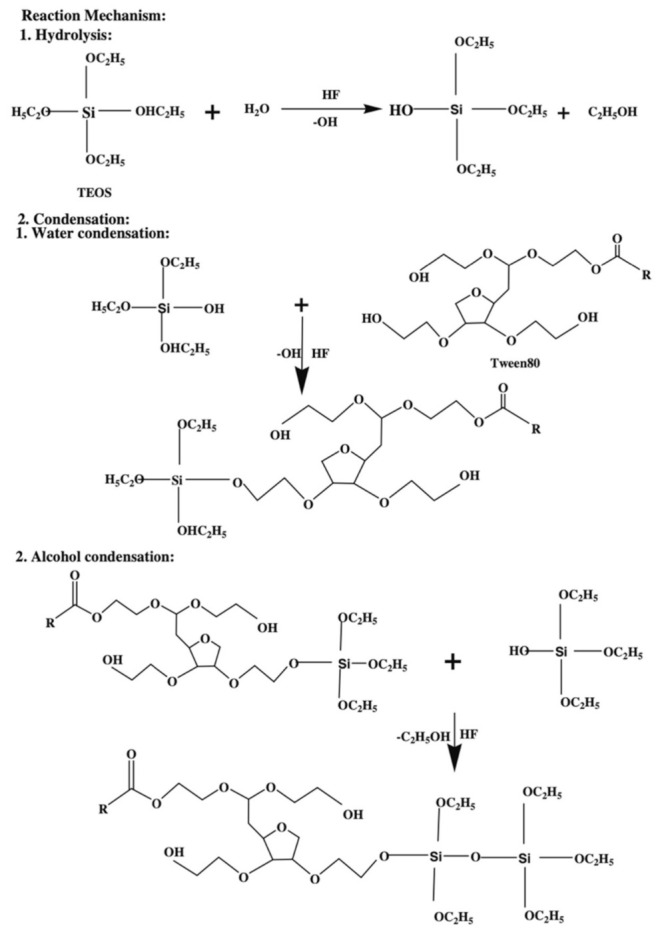
Synthetic route for porous TEOS “wet gel” [43].

The spin-on deposition is particularly suitable where deposition with a good planarization and gap-fill properties are required [8]. Furthermore, the sol-gel spin-on technique has several advantages over CVD because it can produce ILDs with a high degree of porosity which can achieve an extremely low dielectric constant around 2.0 [40,41,42]. The technique is performed by dispensing prepolymer (usually a viscous liquid) on the center of a spinning substrate. The centrifugal force from spinning would allow the prepolymer to expand uniformly on the substrate. The film thickness, in this case, is controlled by the spinning speed and prepolymer viscosity [8]. After spinning, the viscosity of the prepolymer increases, which will eventually turn into a “wet gel”. The wet gel is an incomplete-rigid structure containing a small amount of solvent or moisture. This is an example demonstrating the formation of TEOS wet gel (Figure 8) [43]. The TEOS molecule undergoes a hydrolysis process with water under acidic conditions producing a hydroxy group attached to the center silicon atom. The hydrolyzed TEOS would later condense with itself and Tween 80 (a porogen) as a pre-polymerized coating solution during the condensation process. This solution is subsequently spun on a precleaned wafer at 3000 rpm for 30 s. After that, the “wet gel” further forms between Si–OH and other free hydroxy groups [44]. The wet gel will undergo thermal treatment, typically below 250 °C, to remove any solvent present in the matrix, followed by a thermally initiated curing step, with temperatures varying from 350 to 600 °C to finish all the cross-linking among molecules. The final curing process can also simultaneously remove any porogen that is existing in the system and create a thin layer of porous film [8,43,45]. However, the sol-gel spin-on technique is not an efficient deposition method for mass production. During a dual-damascene integration process, many layers of different electrical components will be deposited on top of each other. The engineering of a production line that can switch between spin-coating and various CVD chambers is extremely complicated and expensive. Furthermore, the high temperature from the final thermal annealing step will inevitably damage other electronic components.

### 3.2. Chemical Vapor Deposition (CVD)

While the spin-on technique is simpler, inexpensive to implement, and can benefit from the engineering of molecules and polymers made by chemists, the CVD (often enhanced by a plasma known as PECVD), is a standard in the field of dielectric deposition in micro-electronics and is often preferred by manufacturers [46,47]. The industry favors fabricating ILDs with the CVD method for several reasons. First, the film made by this method has a structure that resembles the structure of monocrystalline silicon (better conformality), which is capable of filling small gaps. Second, this technique can achieve sub-20 nm level film thickness, which can provide higher wiring density and complexity. Furthermore, such a technique can easily be adapted to industrial mass production without excessive modifications of the existing production lines. The fundamental principles of CVD involve gas-phase reaction chemistry, thermodynamics, heat and material transfer, fluid mechanics, surface and plasma reactions, thin-film growth mechanism, and reactor engineering [8]. During the PECVD process, a significant percentage of precursor atoms and molecules within the carrier gas is ionized and oxidized inside the chamber (Figure 7b). These gas-phase active silane intermediates flow on the surface of the substrate and proceed with a series of chain-branch reactions [48,49,50]. The main advantage of plasma enhancement is that the electrons created from ionization are extremely light compared to the weight of the atoms. This makes the energy exchange between electrons and neutral gas very inefficient, which, in turn, enables the electrons to maintain a very high energy level (equivalent to the energy level at thousands of kelvins), while the neutral atoms remain at the ambient temperature. Such characters allow PECVD to be the most suitable deposition technique for fabricating films onto substrates containing metal layers or other temperature-sensitive structures.

## 4. Integration Challenges

The replacement of silicon dioxide in large-scale integrated (VLSI) interconnected dielectrics (with low-k and pULK) added significant complexity to the integration of the interconnected structure. While it is relatively easy to fabricate porous thin films with a dielectric constant lower than 2.4, the real challenge is successfully integrating the fabrication of the thin film into the current chip manufacturing process. Porous ILDs, unlike SiO_2_, have low mechanical strength, thermal stability, and adhesive strength [15,27]. Furthermore, their porous structure makes them tend to trap other chemicals. As said by Thomas Abell, the integration of low-k materials is comparable to building a fire and waterproof wall out of sponge rather than concrete [15]. All the physical features of porous low-k dielectrics bring challenges to latter integration processes.

### 4.1. Improving Adhesion

The first challenge of the integration is strengthening the pULK layers’ interfacial adhesion. The stickiness of the porous dielectric layer is relatively low. This is caused by a high carbon concentration during the PECVD process. To eliminate such a problem, a transition layer that chemically resembles the pULK layer with improved adhesiveness must be present on the surface of the dielectric layer. Grill et al. reported a process where the precursors for the fabrication of the ILD were introduced gradually into the PECVD reactor while an oxygen-containing plasma was already active in the chamber [27]. After the completion of the initial oxidized layer, the oxygen flow was then reduced, and the precursor flow was adjusted to the regular deposition flow of the bulk ILDs. This process created an initial oxide layer in contact with the dielectric cap (SiCNH), followed by a smooth graded layer where the C concentration increased from almost 0% to its concentration in the bulk ILD [27,51]. Making such an interlayer only requires changing deposition parameters of the CVD chamber, but the parameter adjustment becomes more complicated for the pULK film with subtractive porosity (SiCOH precursor + porogen precursor). The detailed description can be found in the published patents [52,53].

### 4.2. Dual-Damascene Process and Plasma-Induced Damage

**Figure 9:**
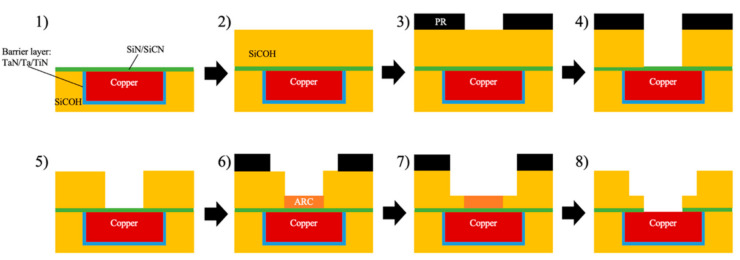
Dual-Damascene process flow for Cu/low-k interconnects [11].

Plasma technology has been very prevalent in micro-electronic manufacturing processes, including cleaning, surface treatment, dielectric etching, metal barrier deposition, and metal deposition [11]. One important point is that these are post-fabrication plasma treatments, which are not part of the thin film fabrication process. The degradation of porous ILDs caused by the plasma-related treatment is known as Plasma-Induced Damage (PID). Compared to crystalline silicon dioxide, pULK materials’ spongy nature has made them mechanically weak, thermally unstable, and hydrophilic. To better understand where PIDs come from, it is necessary to know the basic integration process known as the dual-damascene process. The first task is to incorporate copper into the system. The IC industries replaced aluminum wiring with a lower resistance copper to minimize the resistance-capacitance (RC) delay of transistors [54,55]. The replacement of aluminum introduced more complications on the damascene process because copper is not suitable for reactive ion etching due to the low volatile by-products that can diffuse into the pores from the ILDs. To solve this problem, a Cu barrier layer, usually TiN [56], TaN, or Ta, is deposited onto the surface of the dielectric layer prior to the deposition of copper (blue portion in Figure 9). The Cu barrier layer is deposited by physical vapor deposition (PVD) with sputtering enhanced by plasma. After this step, a Cu seed layer is also deposited with the same method, followed by electrochemical plating (ECP) of Cu [57,58]. After Cu deposition, chemical mechanical polishing (CMP) was performed on top to grind down the extra portion of Cu and expose the porous dielectric layer [59,60]. An NH_3_ or H_2_ plasma clean is performed to remove copper oxides prior to the deposition of the cap layer for adhesion improvement [61,62]. Both of these steps can cause PID on the pULK layer. Another pULK layer will be deposited on top of the cap layer for the subsequent patterning of the copper wire. O_2_ plasma is widely used during the photoresist (PR) patterning process, and the presence of oxygen radicals can easily damage the low-k dielectric film’s surface. To limit the PID from this process, H_2_ can also be used as an alternative plasma gas to replace O_2_ [63,64]. After the removal of the photoresist layer, an anti-reflective coating (ARC) material is filled in the gap between dielectric layers with the spin-on method to protect the silicon nitride layer. This material can later be removed as a part of the photoresist stripping process [65]. The deposition cycle can be repeated for multi-layer fabrications.

### 4.3. PID Prevention

Over the past two decades, while the dielectric constant keeps getting lower, the integration challenges, such as PID, motivate the innovation of precursors and deposition techniques. Various modifications of silane and siloxane monomers and polymers have enabled the ILDs to maintain low dielectric constants, high breakdown voltages, good thermal stabilities, and better hydrophobicity to meet the critical requirements of plasma-involved integration.

**Figure 10:**
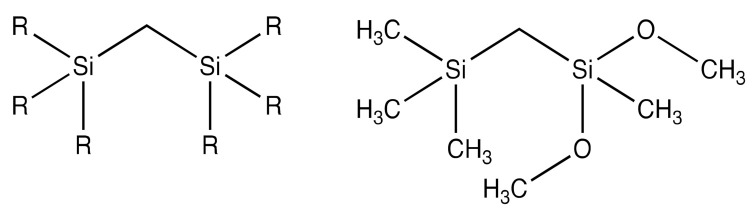
Schematic structure of a Si–C–Si precursor (**left**) and TDMMS (**right**) as a typical example.

As discussed in the previous section, plasma-induced damage (PID) presents everywhere in the dual damascene process. The harsh conditions of plasma would cause a depletion of carbon atoms on ILD’s contacting layer with conductive materials. The PID layer is a hydrophilic low-carbon-containing SiO_2_-like dense layer. As the transistor size shrinks, the PID layer occupies a larger portion within pULK ILD, which brings down the performance of the chips. To fabricate a functional pULK ILD at 32 nm technology node or smaller, the PID layer must be kept at a very small portion of the ILD layer. This means sacrificial carbon content must increase within precursors while maintaining a relatively high silicon ratio. To meet this standard, Si–C–Si structured precursors, such as [trimethylsilylmethyl]dimethoxymethylsilane (TDMMS), are mixed with conventional SiOCH precursors to produce pULK ILDs. Figure 10 shows a general schematic structure of Si–C–Si precursors and the chemical structure of TDMMS. Compared to carbon-rich precursors with a single silicon atom per molecule, the Si–C–Si structured precursors have two silicon atoms per molecule, which can produce ILDs with higher SiO_x_ crosslinking density while maintaining a relatively high carbon content [27]. In Table 5, the PID ratios for V2 and V4, within the similar dielectric constant range, are significantly lower than those of V1 and V3, suggesting that the increase of Si–C bond densities can provide better plasma-resistance for dielectric interlayers.

**Figure 11:**
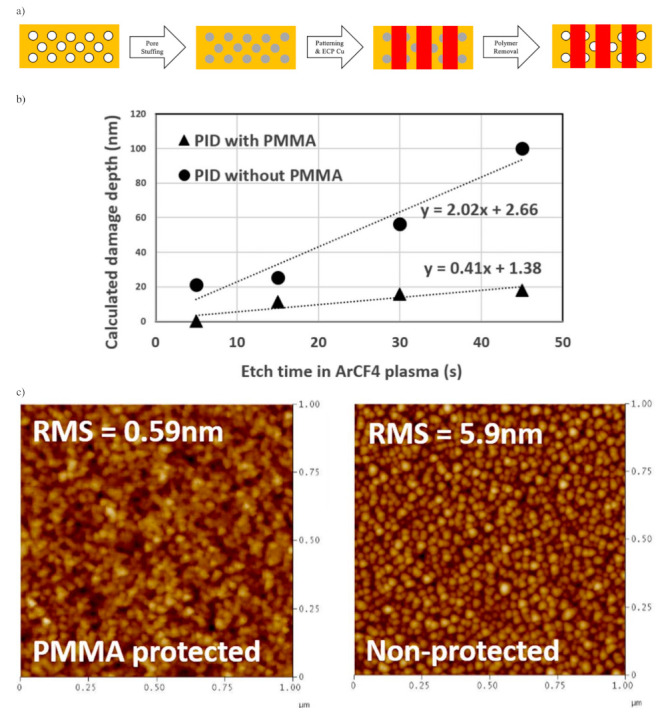
(**a**) Schematic description of the dual damascene process via P4 approach; (**b**) Penetration of Ar/CF4 plasma-induced damage for both stuffed and non-stuffed p-OSG; (**c**) Surface roughness by AFM for stuffed and non-stuffed samples after Ar/CF4 plasma etch. Note that stuffed sample received additional thermal un-stuffing process before AFM characterization.

Though a highly porous structure could be obtained after PECVD, too many pores can also be problematic. First, the copper substrate, unlike the older generation Al substrates, tends to diffuse inside the pores, causing the ILDs’ dielectric constant to rise. Furthermore, as pore size gets larger, the film tends to trap moisture inside pores leading to higher conductivity. Furthermore, during the ECP of Cu, atoms will also inevitably diffuse inside pores on the ILDs’ surface and compromise their dielectric properties. The copper barrier layer and etching stop layer from previous sections are the two types of dense protection layers for stopping atom-diffusion. The atomic layer deposition (ALD) of tantalum or titanium-based materials, such as Ta and TaN or TiN, was considered as one of the most promising ways to stop metallic diffusion [8]. Yet, the gaseous TaN during ALD deposition of such materials can penetrate into the pores on the dielectric surface as well, which compromises the conductivity of the previously fabricated ILDs. One promising way to stop such undesirable penetration is sealing pores from the surface region to create a dense “skin layer”. According to Puyrenier et al., the composition of such a layer can be varied by different plasma treatments: He, NH_3_/N_2_, etch + ash, or NH_3_ plasma treatment [9]. Specifically, NH_3_ plasma treatment induces a thin top layer with the lowest permeability coefficient; thus, this plasma is the most efficient for surface pore sealing (Table 8). In 2007, Peng and coworkers successfully applied NH_3_ plasma treatment on MSQ porous ILD to create a very thin SiCN layer (~10 nm) with little damage to the ~300 nm thick pULK film. They achieved the best results in terms of mechanical strength and anti-diffusion properties at 300 °C for 10 s. The depth/thickness of the sealed layer is dependent on the temperature and duration of the plasma treatment [10]. Besides NH_3_ plasma treatment, post porosity plasma protection (P4) or pore stuffing has also gained a lot of attention during recent years. The filling materials are proprietary methacrylate-based copolymers such as poly(methyl methacrylate) (PMMA) [66]. The schematic description of the P4 process is shown in Figure 11a. A layer of pULK film is initially deposited on a substrate with the PECVD method. After fabrication, a CO_2_ plasma treatment is used to activate the low-k surface and avoid polymer de-wetting. The PMMA polymer would be spin-coated on the top surface of the dielectric layer. By thermal annealing well above the glass transition temperature of the polymer, the solvent would further evaporate, and polymers would also simultaneously penetrate into the pULK film. By optimizing thermo-induced pore stuffing and propylene glycol monomethyl ether acetate (PGMEA) rinse processes, uniformly stuffed low-k film was obtained. After the photoresist patterning process and deposition of the metal barrier layer and Cu conductors, the PMMA polymer is burned out by thermal annealing at 450 °C in N_2_ [67]. As shown in Figure 11b, the calculated damage depth suggests that PID for ILD without P4 treatment is significantly higher than that of P4 treated ILD. This effect is even more prominent as the exposure time of Ar/CF_4_ etching gets longer. Based on the atomic force microscopy (AFM) characterizations shown in Figure 11c, the surface roughness for a stuffed sample is ten times smaller than that of non-protected samples. This enables better continuity with a very thin barrier layer, and hence, less metal diffusion [67].

## 5. Conclusions

To improve the performance of ICs, the dielectric materials constantly evolved to achieve a lower dielectric constant. This review covers the evolution of organosilicon ILDs through three aspects, classification of different types of organosilicon dielectric materials and their precursors, ILD fabrication techniques, and integration challenges and possible solutions during the fabrication process. At first, the shrinkage of the microprocessors’ size in electronic devices puts a high demand on reducing the dielectric constant of interlayer dielectric materials. The reduction of the dielectric constant of SiO_2_ allowed the invention of newer dielectric materials such as SSQ, FSG, and SiCOH. Within the SSQ materials category, MSQ outperforms other similar SSQ materials due to its high thermostability, good metal adhesion, relatively high hydrophobicity, and low dielectric constant (2.7–2.9). However, the hardness of such a material is far less than that of SiO_2_, which makes it a less desirable candidate for ILD applications. FSG materials, despite having good dielectric property (3.2–4.0) and hydrophobicity, cannot tolerate high temperatures due to thermally unstable Si–F bonds. Carbon doped silica or SiCOH materials have a low dielectric constant (2.7–3.3) and better hardness over other materials, especially the SiCOH ILDs made with a DEMS precursor, which appeared to have unexpectedly good mechanical and dielectric properties. Nevertheless, these dense materials’ dielectric constant bottlenecks at 2.6. As the IC size kept shrinking, ILD materials with even lower dielectric constant were demanded. pULk materials were discovered to overcome this bottleneck. Meanwhile, the low mechanical and thermal stability of pULk materials brings many new challenges for the dual-damascene integration process; therefore, lots of re-engineering for the integration process has advanced during the past few decades to maximally preserve pULK films’ dielectric properties. The adhesion is improved by applying oxygen plasma at the initial deposition phase to create an initial oxidized state, which generates better stickiness. Methods such as increasing carbon content, surface NH_3_ plasma treatment, and P4 treatment were invented to minimize the integration-related plasma-induced damage. In the near future, the dimensions of porous ILDs will keep shrinking down to few nanometers, and the electrical, mechanical and thermal stability requirements will keep getting higher. Novel organic-inorganic nanocomposites and 3D porous materials, as well as engineering improvements, are urgently needed to pave the road for the next generation of IC packing.

## Figures and Tables

**Table 1:** Dielectric constants of various contemporary low-k materials [7].

Classification	Material	Fabrication	Dielectric Constant (k)
Silicon dioxide	SiO_2_	CVD	3.9–4.5
Silsesquioxane-based	Hydrogen–Silsesquioxane (HSSQ)	Spin-on	2.9–3.2
	Methyl–Silsesquioxane (MSSQ)	Spin-on	2.6–2.8
Silica-based	FSG	CVD	3.2–4.0
	SiCOH	CVD	2.7–3.3
Porous	Porous HSSQ	Spin-on	1.7–2.2
	Porous MSSQ	Spin-on	1.8–2.2
	Porous SiCOH	Spin-on/CVD	1.5–2.5
Air gaps	Air	-	1.0

**Table 2:** Molar ratio of Si–C, Si–O, Si–H bonds with Si within typical organosilicon precursors.

Molecule	Si–C:Si	Si–O:Si	Si–H:Si	Structure
DEMS	1:1	2:1	1:1	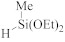
MTES	1:1	3:1	1:1	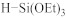
DMOMS	1:1	2:1	1:1	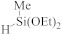
TOMCATS^®^	1:1	1:1	1:1	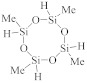
OMCTS	2:1	1:1	-	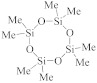
DMDOSH	2:1	2:1	-	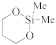
DMDMOS	2:1	2:1	-	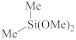
3MS	3:1	-	1:1	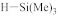

**Table 3:** The preparation conditions and dielectric properties of ILDs made with DEMS, 3MS, and DMDMOS precursors [22].

Classification	DEMS	3MS	DMDMOS
RF Power	300	600	450
Dielectric constant	2.90	2.85	2.88
Precursor flow rate	1000 (mg/min)	540 (sccm)	1500 (mg/min)
He (sccm)	150	N/A	500
O_2_ (sccm)	0	90	50
CO_2_ (sccm)	0	N/A	N/A
N_2_O (SCCM)	N/A	N/A	0
Young’s modulus (GPa)	16.5	8.76	6.68
Hardness (GPa)	2.8	1.44	1.2

**Table 4:** SiCOH precursors with embedded porogen organic groups.

Name	Porogen Functional Group	Structure
vinylmethyldiethoxysilane	alkene group	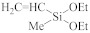
vinyltriethoxysilane	alkene group	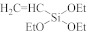
vinyldimethylethoxysilane	alkene group	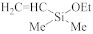
cyclohexenylethyltriethoxysilane	cyclohexene group	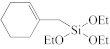
1,1-diethoxy-1–Silacyclopent-3-ene	silacyclopent-3-ene	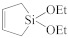
divinyltetramethyldisiloxane	alkene group	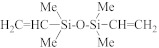
2-(3,4-epoxycyclohexyl) ethyltriethoxysilane	epoxy group	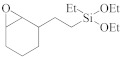
2-(3,4-epoxycyclohexyl) ethyltrimethoxysilane	epoxy group	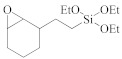
hexavinyldisiloxane	alkene group	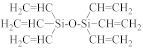
trivinylmethoxysilane	alkene group	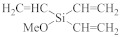
Trimethylsilyloxy-acetylen	alkyne group	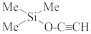
1-(trimethylsiloxy)-1,3-butadiene	1,3-butadiene	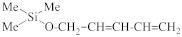
di-t-butoxydiacetoxysilane	carboxyl group	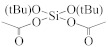

**Table 5:** Comparison among pULK ILDs fabricated with different precursors [27].

Classification	DEMS + BCHD			DEMS + BCHD + TDMMS			Embedded Porogen	Embedded Porogen + TDMMS	
Batch code	V1.a	V1.b	V1.c	V2.a	V2.b	V2.c	V3	V4.a	V4.b
k (@150 °C)	2.53	2.4	2.2	2.56	2.35	2.2	2.46	2.53	2.42
Breakdown voltage (MV/cm)	>7.3	>6.0		>6.5	>6.0		>7.0	7.0	
Elastic modulus (GPa)	7.2	4.9	4.7	6.5	4.73	3.04	6.64	10.2	6.64
Adhesion (J/cm^2^)	4.5	4.4					3.9	4.4	3.9
Carbon percentage (%)	15.7	15.5		20.6	23.2	35.0	21.1	16.3	17.4
Porosity (%)	16.3	24.5	29	19	22	26.5	14.4	17.9	19.7
Pore diameter (nm)	1.2	1.2	2.4	1.2	1.2	1.32	1	1.1	1.3
PID (ratio control)	0.67	0.9	1	0.57	0.55	~0.5	0.57	0.49	0.65

**Table 6:** Results of matrix/porogen ratio and dielectric constant of the three deposited films before and after porogen removal [33].

Molecule	Structure	As Deposited after 390 °C		Anneal under N_2_	
		Matrix %/Porogen %	k (Hg 25 °C)	Matrix %/Porogen %	k (Hg 25 °C)
1-Hexene	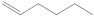	82/18	2.8	92/8	2.3
Hexadiene	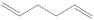	69/31	2.8	91/9	2.4
Cyclohexene oxide	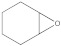	75/25	2.6	89/11	2.2

**Table 7:** Performances of pULK dielectric films from DEMS with ATRP and BCHD [25].

**pULK Performance (I)**							
**Porogen**	**Thickness (Å)**	**Uniformity** **(1 Sigma%)**	**Stress (MPa)**	**Shrinkage (%)**	**Shrinkage** **Uniformity**	**k Value**	**Refractive Index**
BCHD	4930	1.4	50	17.2	3.44	2.48	1.355
ATRP	4920	1.3	60	15.4	3.40	2.59	1.335
**pULK Performance (II)**							
**P** **orogen**	**Pore Size (Å)**	**Porosity (%)**	**Hardness (GPa)**	**Young’s** **Modulus (GPa)**	**Adhesion** **(J/m^2^)**	**Deposition** **Rate (Å/min)**	**SiCH_3_/SiO (Area)**
BCHD	11.8	25	0.7	5.1	5.4	6000	3.6
ATRP	9.5	23	1.0	7.1	5.9	4000	2.6

**Table 8:** Thickness and permeability coefficients of the modified top layer.

Samples	Surface Layer Thickness Measured by Ellipsometry (nm)	Surface Layer Permeability Coefficient (mol m^−1^ s^−1^ Pa^−1^)
Low-k	N/A	N/A
Low-k + He	9 ± 1	5 × 10^−17^
Low-k + NH_3_/N_2_	6 ± 1	9 × 10^−17^
Low-k + etch + ash	9 ± 1	7 × 10^−20^
Low-k + NH_3_	8 ± 1	8 × 10^−21^
